# Metformin use and preeclampsia risk in women with diabetes: a two-country cohort analysis

**DOI:** 10.1186/s12916-024-03628-0

**Published:** 2024-09-27

**Authors:** Hannah Gordon, Noor Salim, Stephen Tong, Susan Walker, Manarangi De Silva, Catherine Cluver, Parinaz Mehdipour, Richard Hiscock, Lauren Sutherland, Ann Doust, Lina Bergman, Anna-Karin Wikström, Anthea Lindquist, Susanne Hesselman, Roxanne Hastie

**Affiliations:** 1 Department of Obstetrics, Gynaecology and Newborn Health, University of Melbourne, Melbourne, Australia; 2 Mercy Perinatal, Mercy Hospital for Women, Heidelberg, VIC Australia; 3 Department of Women’s and Children’s Health, Uppsala University, Uppsala, Sweden; 4 Centre for Clinical Research, Uppsala University, Falun, Sweden; 5 Department of Obstetrics and Gynecology, Stellenbosch University, Cape Town, South Africa; 6 Centre for Reproductive Health, Institute for Regeneration and Repair, University of Edinburgh, Edinburgh, UK; 7 Department of Obstetrics and Gynecology, Institute of Clinical Sciences, Sahlgrenska Academy, University of Gothenburg, Gothenburg, Sweden; 8 Department of Obstetrics and Gynecology, Sahlgrenska University Hospital, Region Västra Götaland, Gothenburg, Sweden

**Keywords:** Metformin, Pregnancy, Gestational diabetes, Pre-eclampsia, Gestational hypertension, Pregnancy-induced hypertension

## Abstract

**Background:**

Metformin is a hypoglycaemic medication that has been proposed to treat or prevent preeclampsia. Combining national birth data from Scotland and Sweden, we investigated whether metformin used during pregnancy was associated with an altered risk of developing a hypertensive disorder of pregnancy.

**Methods:**

We utilised data from two population-based cohorts: Scotland (2012–2018) and Sweden (2007–2019). Nulliparous women with gestational diabetes or type 2 diabetes who had birth outcome data linked with medications prescribed during pregnancy were included. The association between metformin prescription and hypertensive disorders of pregnancy was characterised using inverse probability weighted regression analysis, adjusting for variables that predict metformin use and potential confounders. Adverse neonatal outcomes were included as secondary outcomes. Results from both countries were then combined in a meta-analysis using a random effects model.

**Results:**

The Scottish cohort included 3859 women with gestational diabetes or type 2 diabetes. Of these women, 30.8% (*n* = 1187) received at least one metformin prescription during pregnancy. For Sweden, 7771 women with gestational diabetes were included where 19.3% (1498) used metformin during pregnancy. Metformin prescription was not associated with an altered risk of any hypertensive disorder of pregnancy (Scotland adjusted relative risk (aRR) 0.88 [95% confidence interval (CI) 0.66–1.19]; Sweden aRR 1.08 [95% CI 0.86–1.37]) or preeclampsia (Scotland aRR 1.02 [95% CI 0.66–1.60]; Sweden aRR 1.00 [95% CI 0.72–1.39]). Combining adjusted results in a meta-analysis produced similar findings, with a pooled RR of 0.98 (95% CI 0.79–1.18) for any hypertensive disorder and RR 1.01 ([95% CI 0.73–1.28]) for preeclampsia. For neonatal outcomes, metformin was associated with a reduced risk of birthweight > 4500 g in Scotland (aRR 0.39 [95% CI 0.21–0.71]) but not in Sweden. There was no association between metformin and preterm birth or birthweight < 3rd or < 10th percentiles. Pooling results from both countries, metformin was not associated with adverse neonatal outcomes, including preterm birth (RR 1.00 [95% CI 0.89–1.13]), and birthweight < 10th percentile (RR 0.82 [95% CI 0.60–1.13]) or < 3rd percentile (RR 0.78 [95% CI 0.41–1.48]).

**Conclusions:**

In this two-country analysis, metformin use in pregnancy among women with diabetes was not associated with an altered risk of developing any hypertensive disorder of pregnancy. In the combined meta-analysis, metformin was not associated with an altered risk of adverse neonatal outcomes.

**Supplementary Information:**

The online version contains supplementary material available at 10.1186/s12916-024-03628-0.

## Background

Preeclampsia is a hypertensive disorder of pregnancy and a leading cause of maternal and neonatal morbidity and mortality [1]. Currently, there are no medical therapeutics available to treat preeclampsia. Aspirin is the only known preventative agent [2], which reduces the risk of preterm preeclampsia (preeclampsia with delivery < 37 gestational weeks) by 62% (OR 0.38, [95% CI 0.20–0.74]), but not preeclampsia occurring at term gestations (OR 0.95 [95% CI 0.57–1.57]) [3]. Term preeclampsia is far more common, and is associated with at least the same maternal and perinatal morbidity as preterm preeclampsia due to its prevalence compared with preterm disease [4]. Yet, there are currently no preventative agents for term preeclampsia.

Metformin has been proposed as a potential treatment for preeclampsia, with preclinical studies demonstrating effects on placental and maternal vasculature, including increased angiogenesis, reduced endothelial dysfunction and potential downregulation of mitochondrial electron transport chain activity [5]. A randomised trial of 180 women with preterm preeclampsia (diagnosed prior to 32 weeks’ gestation) reported that compared with placebo, 3 g of oral metformin was associated with a non-significant prolongation of pregnancy of 7.6 days, with a further prolongation of pregnancy (median 17.5 days) observed in women who continued to take metformin throughout the study period [6, 7].

A meta-analysis of randomised controlled trials found metformin was associated with a lower risk of developing preeclampsia, compared with insulin (RR 0.68 [95% CI 0.48–0.95]) [8]. However, to our knowledge, there are no randomised controlled trials comparing metformin with placebo/control in a diabetic population. Observational studies comparing metformin with control are inconclusive, limited by predominantly small cohorts, and typically reporting pre-eclampsia as a secondary outcome [8–10]. Interpreting findings is additionally difficult due to the varied cohorts of women, including those with diabetes, polycystic ovarian syndrome and a high body mass index [8, 9, 11].

Given the lack of studies specifically aimed at investigating the association between metformin and hypertensive disorders of pregnancy, we set out to examine this in a two-country analysis using national level data from Scotland and Sweden.

## Methods

We conducted two register-based cohort studies using population-based data from Scotland and Sweden and then pooled findings from both countries in a meta-analysis.

### Study population

For Scotland, nulliparous women with a singleton pregnancy and diagnosis of gestational diabetes or Type 2 diabetes with a birth recorded in the Scottish Morbidity Record from 2009 and 2018 were included. Women with Type 2 diabetes were included in this study due to the low uptake of screening for gestational diabetes in Scotland, as risk factor-based screening is routinely performed, rather than universal screening [12]. The Scottish Morbidity Records hold data relating to pregnancy, birth and perinatal outcomes for all women and infants discharged from maternity hospitals across Scotland. This dataset is regularly audited for quality control [13] and is validated against the National Records of Scotland to ensure completeness [14].

For Sweden, nulliparous women with a singleton pregnancy and a diagnosis of gestational diabetes with a birth recorded in the Medical Birth Register from 2007 to 2019 were included. The Medical Birth Register captures 98% of births across Sweden, providing very high-quality population-level perinatal data [15]. These data were linked with the National Patient Register, the Swedish Prescribed Drug Register and the Education Register held by Sweden Statistic to provide detailed pregnancy, birth, maternal education and medication prescription information [16–18]. There is no national consensus guideline for diagnosis of gestational diabetes in Sweden, with screening approaches varying according to local hospital guidelines [19].

### Exclusion criteria

Participants were excluded if they had a recorded diagnosis of Type 1 diabetes or if the timing of diabetes diagnosis was unclear or missing. Pregnancies missing gestational age at birth were also excluded. To account for maternal clustering, and the increased risk of preeclampsia among primiparous women [20], we excluded multiparous women.

### Exposure

Maternal metformin use was identified through the Scottish National Prescribing Information System (Scotland) and the Swedish Prescribed Drug Register (Sweden) and defined as a prescription dispensed during pregnancy (Scotland), or a prescription dispensed during pregnancy or in the 3 months prior to conception (Sweden). To minimise the number of women with isolated preconception exposure in the Swedish cohort, women with pre-existing Type 2 diabetes were excluded from the Swedish analysis.

### Primary outcome

Diagnosis of hypertensive disorders of pregnancy, including preeclampsia and pregnancy-induced hypertension, were identified by International Classification of Diseases (ICD) coding (ICD codes O11.1–5, O11.9, O14.0–2, O14.9 and O15.0–2, O15.9).

### Secondary outcomes

Secondary outcomes included preterm birth (< 37 weeks’ gestation), neonatal intensive care unit admission, major congenital anomaly, perinatal death, macrosomia (> 4500 g), birthweight < 10th percentile and < 3rd percentile (Table S1). Neonatal birthweight centiles were calculated according to INTERGROWTH 21st percentiles [21], adjusted for infant sex and gestational age at birth. Outcomes occurring in fewer than five women or neonates in the Scottish cohort were not reported to maintain patient confidentiality.

### Covariates

Covariates predicting treatment assignment (selection model) and potential confounding variables (outcome model) were determined a priori by the authorship team and with the use of Directed Acyclic Graphs (Additional file 1: Fig. S1, S2) [8, 22–25].

Covariates included in the selection model were year of birth, maternal age, smoking during pregnancy, maternal BMI, socioeconomic status (Scotland only), highest level of maternal education (Sweden only), maternal country of birth (Sweden only), conception via in vitro fertilisation (IVF) (Sweden only), and pre-existing maternal medical conditions.

For the outcome model, covariates included maternal age, smoking during pregnancy, BMI, socioeconomic status (Scotland only), pre-existing maternal medical conditions, highest level of maternal education (Sweden only), maternal country of birth (Sweden only) and IVF pregnancy (Sweden only). The same covariates were used in the models for each secondary outcome, with insulin included as an added covariate in the outcome model for the Swedish population.

Pre-existing medical conditions in the Scottish cohort were identified in the Scottish Morbidity Record via ICD coding and included chronic hypertension and polycystic ovary syndrome (Scotland). Pre-existing medical conditions in the Swedish cohort included chronic hypertension, kidney disease, and systemic lupus erythematosus and were identified by checkboxes in the Medical Birth Register (Table S1). Differences between included covariates for Sweden and Scotland relate to data availability.

### Handling of missing data

Data were largely complete (> 90%) across relevant demographic and clinical characteristics in the cohorts. Ethnicity was an exception with 23% missing from the Scottish cohort. For both countries, a complete case analysis was performed.

### Statistical analysis

An a priori statistical analysis plan was developed and agreed upon by the authorship team (Additional File 1). Clinical and demographic characteristics of the population were summarised by metformin use during pregnancy (exposed/unexposed).

To investigate the association between metformin prescription and hypertensive disorders of pregnancy, a doubly robust inverse probability regression adjusted model was employed. This model combines an inverse probability weighted selection model (with variables that predict treatment assignment included) with a regression adjustment (including potential confounding variables).

After adjustment, each covariate was assessed for balance between exposure groups, with a standardised mean difference less than or close to 0.1 deemed adequate (Table S2) [26]. Unadjusted and adjusted results were presented as relative risk with corresponding 95% confidence intervals (95% CIs).

A sensitivity analysis was performed for the Scottish cohort assessing the risk of hypertensive disorders of pregnancy between women with two or more prescriptions dispensed during pregnancy compared with women with diabetes not using metformin. The same data were not available for the Swedish cohort.

Primary and secondary outcomes from Swedish and Scottish cohorts were combined using meta-analysis with a random-effects model to account for the unobserved heterogeneity in the outcomes related to different countries. Non-symmetric confidence intervals underwent log transformation to reduce skewness. Given there were only two included studies, a decision was made not to pool results in a meta-analysis where significant discrepancies between point effects were noted (e.g., macrosomia > 4500 g).

All data were analysed using Stata (version 18.0 MP) [27], according to the statistical analysis plan (Additional File 1).

Ethical approval was granted through the Mercy Health Ethics Committee [HREC 2019–005] on 12th November 2019 and the Swedish Ethical Review Authority [2019–04-25] on 28th January 2020. Permission to access and use the data was obtained from the Electronic Data Research and Innovation Service (eDRIS) (Public Health Scotland) and Statistics Sweden. This research adheres to the Strengthening the Reporting of Observational Studies in Epidemiology (STROBE) guidelines (Table S3).

## Results

**Fig. 1:**
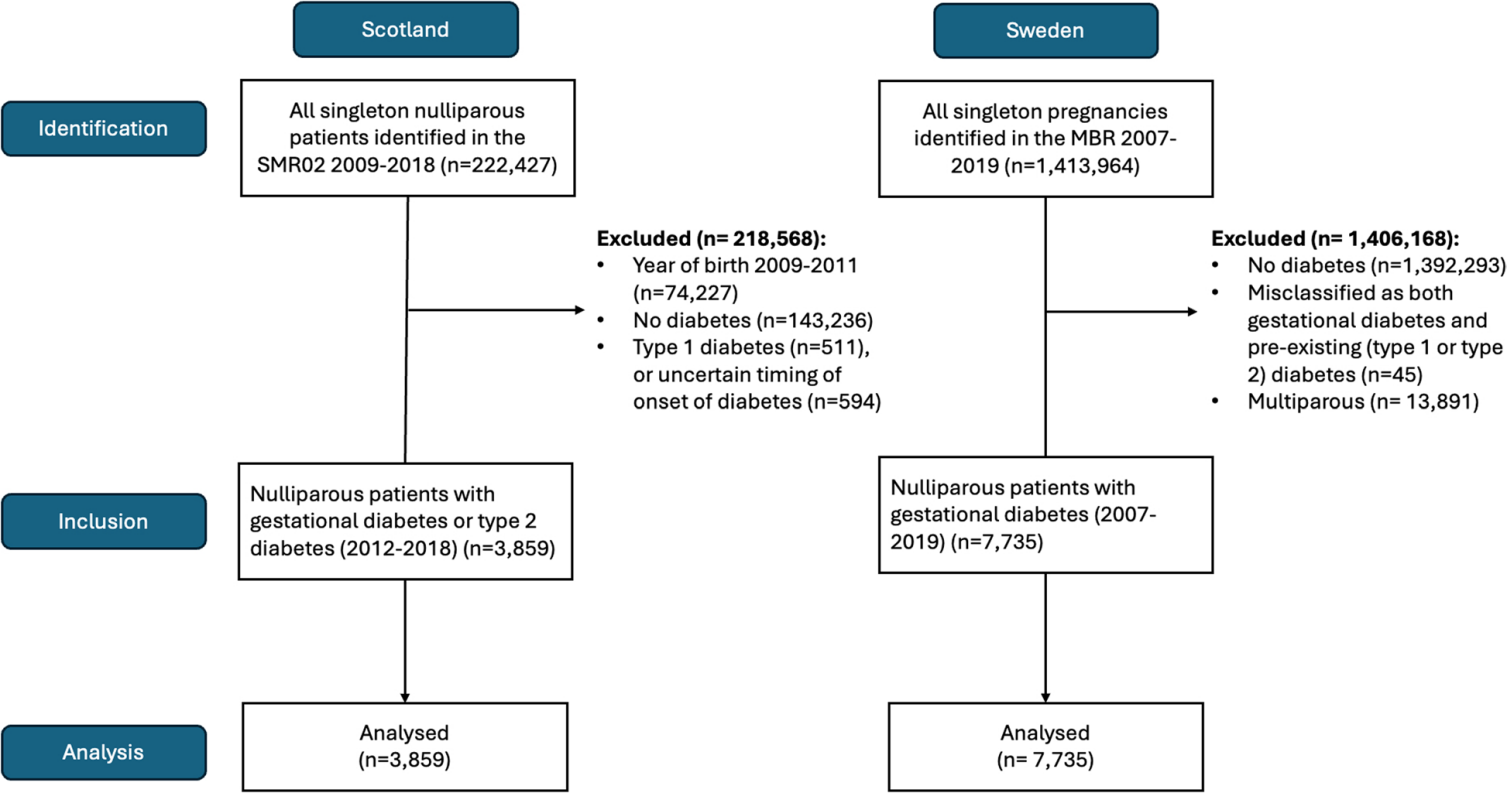
Participant flow diagram

The Scottish cohort included 3859 women with gestational diabetes or type 2 diabetes (Fig. 1), of whom 30.8% (*n* = 1187) had at least one metformin prescription dispensed during pregnancy. For Sweden, 7771 women with gestational diabetes were included, and of these, 19.3% (1498) used metformin during pregnancy.

Among the Scottish cohort, women prescribed metformin during pregnancy were older, more likely to have a BMI 30 kg/m^2^ or greater, identify as ethnically Asian and have polycystic ovary syndrome and/or pre-existing hypertension (Table 1). There was no difference in deprivation score and smoking status between metformin exposed and unexposed individuals.

In the Swedish cohort, there was no difference in maternal age between metformin exposed and unexposed. Women prescribed metformin were more likely to have a BMI 30 kg/m^2^ or greater, less likely to smoke during pregnancy and were more commonly born in Asia or Africa. There was no difference between metformin exposed and unexposed in documented maternal education, conception via in-vitro fertilisation or prevalence of pre-existing medical conditions (hypertension, kidney disease, systemic lupus erythematosus).

**Table 1:** Demographic and clinical characteristics of singleton nulliparous women with diabetes in pregnancy in Scotland and Sweden

**Scotland**	**Metformin use, n **		**Sweden**	**Metformin use, n**	
	**No** ***n*= 2,672**	**Yes** ***n*= 1,187**		**No** ***n*=6,237**	**Yes ** ***n*= 1,498**
**Age in years, **(mean, SD)	30 (5.8)	31 (5.5)	**Age in years, **mean (SD)	30.1 (5.5)	30.4 (5.4)
**Body mass index, **kg/m^2^(mean, SD)	32.4 (7.9)	34.1 (8.0)	**Body mass index, **kg/m^2^(mean, SD)	28.1 (6.2)	31.0 (6.5)
Missing	123 (4.6)	53 (4.5)			
**Body mass index ≥30kg/m**^**2**^,n (%)	1,549 (60.8)	803 (70.8)	**Body mass index ≥30kg/m**^2^, n (%)	2,074 (33.3)	754 (50.3)
Missing	123 (4.6)	53 (4.5)	Missing	296 (4.8)	54 (3.6)
**Ethnicity, **n (%)			**Country of birth,** n (%)		
White	1,775 (87.0)	773 (84.0)	Nordic countries	3,749 (60.1)	746 (49.8)
Multiple ethnic groups	12 (0.6)	6 (0.7)	Europe & North America	575 (9.2)	120 (8.0)
African/Caribbean/Black	50 (2.5)	9 (1.0)	Asia	1,427 (22.9)	432 (28.8)
Asian British/Scottish	174 (8.5)	119 (12.9)	Other	73 (1.2)	33 (2.2)
Other ethnic group	30 (1.5)	13 (1.4)			
Missing	631 (23.6)	267 (22.5)	Missing	413 (6.6)	167 (11.2)
**Deprivation score, **n (%)			**Education, **n (%)		
1 (most deprived)	649 (24.3)	303 (25.5)	University	3,076 (49.3)	689 (46.8)
2	600 (22.5)	278 (23.4)	Secondary school	2,326 (37.3)	562 (37.5)
3	518 (19.4)	246 (20.7)	<12 years of school attendance	662 (10.6)	233 (15.6)
4	532 (20.0)	193 (16.3)			
5 (least deprived)	367 (13.8)	167 (14.1)			
Missing	6 (0.2)	0 (0)	Missing	173 (2.8)	14 (0.9)
**Pre-existing maternal disease, **n (%)			**Pre-existing maternal disease**^a^**, **n (%)	99 (1.6)	22 (1.5)
Chronic hypertension	33 (1.2)	19 (1.6)			
Polycystic ovary disease	10 (0.4)	11 (0.9)			
**Smoking during pregnancy**, n (%)	335 (13.1)	146 (12.8)	**Smoking during pregnancy**, n (%)	405 (6.5)	79 (5.3)
Missing	113 (4.2)	58 (4.0)	Missing	270 (4.3)	73 (4.9)
**Year of birth, **n (%)			**Year of birth, **n (%)		
2012	374 (14.0)	6 (0.5)	2007	356 (5.7)	30 (2.0)
2013	450 (16.8)	8 (0.7)	2008	442 (7.1)	29 (1.9)
2014	500 (18.7)	14 (1.2)	2009	393 (6.3)	26 (1.7)
2015	291 (10.9)	232 (19.6)	2010	413 (6.6)	31 (2.1)
2016	304 (11.4)	300 (25.3)	2011	454 (7.3)	39 (2.6)
2017	344 (12.9)	280 (23.6)	2012	422 (6.8)	49 (3.3)
2018	409 (15.3)	347 (29.2)	2013	389 (6.2)	66 (4.4)
			2014	406 (6.5)	74 (4.9)
			2015	430 (6.9)	108 (7.2)
			2016	424 (6.8)	174 (11.5)
			2017	458 (7.3)	364 (24.3)
			**IVF conception**, n (%)	471 (7.6)	118 (7.9)

*Abbreviations*: *SD* Standard deviation, *IVF* In-vitro fertilisation

^a^Includes chronic hypertension, kidney disease and systemic lupus erythematosus

In Scotland, among women with diabetes, metformin use in pregnancy was not associated with an altered risk of developing a hypertensive disorder of pregnancy in both unadjusted and adjusted estimates (relative risk [RR] 0.90 [95% CI 0.71–1.15]; adjusted RR [aRR] 0.88 [95% CI 0.66–1.19]; 7.8% unexposed *vs* 7.1% exposed), or pregnancy-induced hypertension (RR 0.79 [95% CI 0.57–1.09], aRR 0.80 [95% CI 0.53–1.20]; 5.1% *vs* 4.1%). Similarly, metformin use in pregnancy was not associated with an altered risk of preeclampsia (RR 1.11 [95% CI 0.76–1.63]; aRR 1.02 [95% CI 0.66–1.60]; 2.9% *vs* 3.2%), including preterm preeclampsia (RR 0.83 [95% CI 0.42, 1.64]; aRR 0.81 [95% CI 0.37–1.77]; 1.1% *vs* 0.9%) (Table 2). Results were similar when metformin exposure was considered as two or more prescriptions during pregnancy (Table S4).

**Table 2:** Hypertensive disorders of pregnancy by metformin prescription

	**Scotland**				**Sweden**			
	**No metformin use (2672)**	**Metformin use (*n* = 1187)**	**Relative risk (95% confidence interval)**		**No metformin use (6237)**	**Metformin use (*n* = 1498)**	**Relative risk (95% confidence interval)**	
			**Crude**	**Adjusted**^**a**^			**Crude**	**Adjusted**^**a**^
Hypertensive disorders of pregnancy	209 (7.8)	84 (7.1)	0.90 (0.71–1.15)	0.88 (0.66–1.19)	788 (12.7)	237 (15.8)	1.25 (1.10, 1.43)	1.08 (0.86, 1.37)
Pregnancy-induced hypertension	132 (5.1)	46 (4.1)	0.79 (0.57–1.09)	0.80 (0.53–1.20)	228 (3.7)	94 (6.3)	1.72 (1.36, 2.17)	1.34 (0.98, 1.83)
Preeclampsia	77 (2.9)	38 (3.2)	1.11 (0.76–1.63)	1.02 (0.66–1.60)	566 (9.1)	146 (9.8)	1.07 (0.90, 1.28)	1.00 (0.72, 1.39)
Preterm preeclampsia (< 37 weeks’)	30 (1.1)	11 (0.9)	0.83 (0.42–1.64)	0.81 (0.37–1.77)	124 (2.0)	38 (2.5)	1.01 (0.99, 1.03)	1.00 (0.98, 1.03)

^a^*n* = 3555 in adjusted analysis for Scottish cohort and *N* = 6522 for the Swedish cohort. Adjusted analyses were estimated using a doubly robust inverse probability weighted regression adjustment model

In Sweden, metformin use in pregnancy was associated with an increased risk of hypertensive disorders of pregnancy (RR 1.25 [95% CI 1.10–1.43]; 12.7% unexposed *vs* 15.8% exposed) and pregnancy-induced hypertension (RR 1.72 [95% CI 1.36–2.17]; 3.7% *vs* 6.3%). However, after adjusting, this association was no longer present for hypertensive disorders overall (aRR 1.08 [95% CI 0.86–1.37]) or pregnancy-induced hypertension (aRR 1.34 [95% CI 0.98–1.83]). Metformin in pregnancy was not associated with an altered risk of preeclampsia (RR 1.07 [95% CI 0.90–1.28]; aRR 1.00 [95% CI 0.72–1.39]; 9.1% *vs* 9.8%), including preterm preeclampsia (RR 1.01 [95% CI 0.99–1.03]; aRR 1.00 [95% CI 0.98–1.03]; 2.0% *vs* 2.5%) (Table 2).

**Fig. 2:**
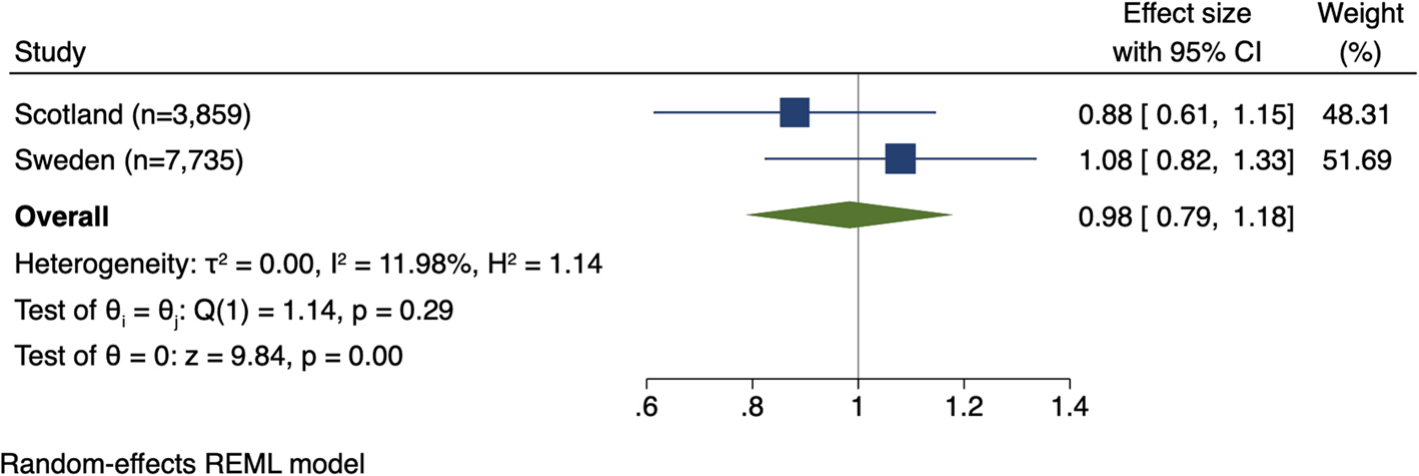
Meta-analysis of metformin use in pregnancy and hypertensive disorders of pregnancy

Next, we combined findings from both countries in a meta-analysis. Pooling adjusted estimates, metformin was not associated with an altered risk of developing a hypertensive disorder of pregnancy (RR 0.98 [95% CI 0.79–1.18]) (Fig. 2, Table 3), preeclampsia (RR 1.01 [95% CI 0.73, 1.28]), preterm preeclampsia (RR 1.00 [95% CI 0.97–1.02]), or pregnancy-induced hypertension (RR 1.05 [95% CI 0.53–1.58]).

**Table 3:** Meta-analysis of metformin prescription in pregnancy and preeclampsia risk

	**Metformin use, *n* (%)**		**Pooled adjusted relative risk (95% confidence interval)**
	**No, *n* = 8909**	**Yes, *n* = 2685**	
Hypertensive disorders of pregnancy	997 (11.2)	321 (12.0)	0.98 (0.79–1.18)
Preeclampsia	643 (7.2)	184 (6.9)	1.01 (0.73–1.28)
Preterm preeclampsia (< 37 weeks’)	154 (1.7)	49 (1.8)	1.00 (0.97–1.02)
Pregnancy-induced hypertension	361 (4.1)	141 (5.3)	1.05 (0.53–1.58)

### Neonatal outcomes

In Scotland, infants exposed to metformin were born at a median (IQR) gestational age of 38 weeks (IQR 37, 39), compared with 38 weeks (IQR 38, 39) among metformin unexposed. Examining neonatal outcomes, we found no association between metformin exposure and adverse outcomes, including preterm birth (< 37 weeks’ gestation) (aRR 0.97 [95% CI 0.94–1.00]; 9.5% unexposed *vs* 11.2% exposed), and neonatal intensive care unit admission (aRR 1.18 [95% CI 0.95–1.46]; 13.6% *vs* 14.1%) (Table 4). Congenital anomalies (*n* = 14) and perinatal death (*n* = 12) were rare outcomes, with numbers in the metformin-exposed group too small to perform further analyses. Investigating birthweight, metformin-exposed infants were 94 g lighter (adjusted mean difference − 94 [95% CI − 134, − 53]) than their unexposed counterparts. Additionally, metformin was associated with a 61% reduction in the risk of macrosomia (birthweight > 4500 g) (aRR 0.39 (95% CI 0.21–0.71]; 2.9% *vs* 1.3%). Investigating small for gestational age infants, metformin was not associated with an altered risk of a birthweight < 10th percentile (aRR 1.00 [95% CI 0.63–1.59; 2.9% *vs* 2.8%) or < 3rd percentile (aRR 0.95 [95% CI 0.39–2.33]; 0.8% *vs* 0.8%).

**Table 4:** Neonatal outcomes by metformin exposure

	**Metformin use, *n* (%)**		**Effect size (95% CI)**^a^	
	**No**	**Yes**	**Crude**	**Adjusted**
**Scotland**	***n* = 2672**	***n* = 1187**		
Preterm birth (< 37 weeks’)	255 (9.5)	133 (11.2)	1.17 (0.96–1.43)	0.97 (0.94–1.00)
Macrosomia > 4500 g	78 (2.9)	15 (1.3)	0.43 (0.25–0.75)	0.39 (0.21–0.71)
Birthweight percentile < 10th^b^	76 (2.9)	33 (2.8)	0.98 (0.66–1.47)	1.00 (0.63–1.59)
Birthweight percentile < 3rd^b^	21 (0.8)	9 (0.8)	0.97 (0.45–2.11)	0.95 (0.39–2.33)
Neonatal intensive care unit admission	365 (13.6)	167 (14.1)	1.09 (0.91–1.30)	1.18 (0.95–1.46)
Missing	65 (2.4)	44 (3.8)		
**Sweden**	***n*** **= 6,237**	***n*** **= 1,498**		
Preterm birth (< 37 weeks’)	638 (10.2)	183 (12.2)	1.19 (1.02, 1.39)	1.13 (0.90, 1.43)
Macrosomia > 4500 g	272 (4.4)	55 (3.7)	0.84 (0.63, 1.12)	0.95 (0.67, 1.35)
Missing	9 (0.1)	1 (0.07)		
Birthweight percentile < 10th	283 (4.6)	67 (4.5)	0.98 (0.76, 1.28)	0.72 (0.50, 1.03)
Missing	19 (0.3)	3 (0.2)		
Birthweight percentile < 3rd	71 (1.1)	16 (1.1)	0.94 (0.55, 1.61)	0.64 (0.26, 1.59)
Missing	19 (0.3)	3 (0.2)		

^a^Effect measure is relative risk for all variables except birthweight which is presented as mean difference. Adjusted analyses were estimated using a doubly robust inverse probability weighted regression adjustment model

^b^Missing, *n* = 13. Not stratified by metformin use due to small numbers

In the Swedish cohort, infants exposed to metformin were born at a median (IQR) gestational age of 39.7 weeks (IQR 38.6, 40.6), compared with 39.3 weeks (IQR 38.3, 40.3) among metformin unexposed. Infants exposed to metformin were at an increased risk of preterm birth (RR 1.19 [95% CI 1.02–1.39]; 10.2% unexposed *vs* 12.2% exposed). However, after adjusting, this association was no longer present (aRR 1.13 [95% CI 0.90–1.43)]. Metformin was not associated with macrosomia (birthweight > 4500 g) (aRR 0.95 [95% CI 0.67–1.35]; 4.4% *vs* 3.7%) or small for gestational age infants, including birthweight < 10th percentile (aRR 0.72 [95% CI 0.50–1.03]; 4.6% *vs* 4.5%) and 3rd percentile (aRR 0.64 [95% CI 0.26–1.59]; 1.1% *vs* 1.1%).

We again pooled adjusted analyses from both countries in meta-analysis, which revealed similar findings. In pooled analyses metformin was not associated with an altered risk of preterm birth (RR 1.00 [95%CI 0.89–1.13]), birthweight < 10th percentile (RR 0.82 [95% CI 0.60–1.13]) or birthweight < 3rd percentile (RR 0.78 [95% CI 0.41–1.48]) (Table 5).

**Table 5:** Meta-analysis analysis of neonatal outcomes by metformin exposure

	**Metformin use, *n* (%)**		**Pooled adjusted relative risk (95% confidence interval)**
	**No, *n* = 8909**	**Yes, *n* = 2685**	
Preterm birth (< 37 weeks’)	893 (10.0)	316 (11.8)	1.00 (0.89–1.13)
Birthweight percentile < 10th	360 (4.0)	102 (3.8)	0.82 (0.60–1.13)
Birthweight percentile < 3rd	92 (1.0)	25 (0.9)	0.78 (0.41–1.48)

## Discussion

Metformin in pregnancy was not associated with an altered risk of developing a hypertensive disorder of pregnancy among women with gestational diabetes or type 2 diabetes in Scotland and Sweden. Importantly, metformin was also not associated with adverse neonatal outcomes in either country. In the Scottish cohort, however, metformin was associated with a 61% reduced risk of macrosomia at birth (> 4500 g), compared with metformin unexposed infants.

Although there is growing interest in the utility of metformin for preventing hypertensive disorders of pregnancy, there are few published studies directly investigating this association. In a meta-analysis of cohort studies, metformin was not associated with an altered risk of developing preeclampsia, compared with control (RR 1.21 [95% CI 0.56–2.61]; 4 studies). These findings were limited by small numbers, with only 51 cases of preeclampsia among 1402 women. Considering randomised controlled trials in this area, metformin has been associated with a significantly reduced likelihood of preeclampsia when examined as a secondary outcome among women with an elevated BMI but without diabetes [28]. Ours is the first study to primarily examine the association between antenatal metformin use and hypertensive disorders of pregnancy. Notably, we did this in not only one, but in two countries and then pooled our findings.

Although we did not find a protective effect of metformin for hypertensive disorders of pregnancy, our results provide reassurance for patients concerned about the potential impact of metformin on neonatal birthweight. In both cohorts, metformin was not associated with adverse neonatal outcomes including birthweight < 10th or < 3rd percentiles, and among the Scottish cohort, metformin was associated with a significant reduction in macrosomia. This may be related to the inclusion of women with type 2 diabetes in the Scottish cohort, at higher baseline risk of large for gestational age [29], and receiving metformin for a longer duration.

Comparatively, the multicentre “MiTy” trial (metformin in women with type 2 diabetes in pregnancy) [30], reported an increased risk of a birthweight < 10th percentile among infants born to women in the metformin arm (13% (*n* = 30) *vs.* 7% (*n* = 15); RR 1.96 [95% CI 1.10–3.64]). Importantly, at the 2-year follow-up, children had no difference in anthropometrics including BMI and mean sum of skinfolds when comparing children exposed to metformin in-utero with those exposed to placebo [31]. Other studies have found conflicting results concerning childhood BMI [32, 33], suggesting the need for further research to better characterise this potential relationship.

### Strengths and limitations

The strength of this study lies in its unique design, a two-country analysis utilising whole country data from Scotland and Sweden. Importantly, both countries hold high-quality data that are routinely audited and capture almost all births [14, 15]. A limitation of our study is that data on patient compliance and metformin doses were not available within our datasets. However, reassuringly, subgroup analysis from the Scottish cohort found similar results when restricting metformin exposure to those who filled two or more prescriptions during pregnancy.

Although metformin did not reduce the risk of developing preeclampsia, most women in our cohort were first prescribed metformin in the third trimester. At this stage, the vascular remodelling associated with preeclampsia has largely occurred (ahead of developing overt disease) [34]. Earlier metformin exposure is likely needed to prevent disease onset. This could be a focus of future studies, with additional stratification of outcomes by diabetes type (type 2 diabetes, gestational diabetes). Induction of labour for diabetes in pregnancy at term is common practice [35]. Comparatively, it may be that induction occurred prior to the onset of diagnosis of preeclampsia in some cases, decreasing the incidence of the disease, and masking any potential benefit of metformin. Despite using data from two population-based cohorts, there were only a small number of women (*n* = 199) who were diagnosed with preterm preeclampsia across both Scotland and Sweden. Thus, large studies in this area are still needed.

There was variation in the screening approaches and diagnostic criteria for gestational diabetes in Scotland and within Sweden. There is no national consensus guideline in Sweden, with different diagnostic criteria and both universal and risk factor-based screening approaches used depending on local hospital guidelines [19]. By comparison, the current Scottish Intercollegiate Guidelines Network recommend a risk factor-based approach to screening for gestational diabetes, with diagnostic criteria in keeping with that proposed by the International Association of the Diabetes and Pregnancy Study Groups [35]. An internal audit of the Scottish Morbidity Records also demonstrates that gestational diabetes is under-recorded [13], resulting in a smaller cohort of patients with diabetes in Scotland than anticipated. Diabetes is independently associated with both metformin use and preeclampsia [36, 37]. We therefore restricted our cohort to women with diabetes to eliminate diabetes status as a confounder. Further research should consider the impact of metformin in women irrespective of their diabetes status.

## Conclusions

In this two-country analysis, metformin was not associated with an altered risk of hypertensive disorders of pregnancy among nulliparous women with gestational diabetes and type 2 diabetes. Importantly, metformin was not associated with an increased risk of adverse neonatal outcomes in either country and in Scotland, metformin use was associated with a reduced risk of macrosomia.

## Supplementary Information

Additional file 1: Statistical analysis plan.

Additional file 2: Figure S1 – Directed acyclic graph of the hypothesised relationship between metformin use in pregnancy and preeclampsia in the diabetic population.

Additional file 3: Figure S2 – Directed acyclic graph of the hypothesised relationship between metformin use in pregnancy and preeclampsia (irrespective of diabetes status).

## Data Availability

The data that support the findings of this study are available from the Electronic Data Research and Innovation Service (eDRIS) (Public Health Scotland), and Statistics Sweden. Restrictions apply to the availability of these data, which were used under license for the current study, and are not publicly available.

## Abbreviations

BMI: Body mass index
aRR: Adjusted relative risk
ICD: International Classification of Diseases
IVF: In vitro fertilisation
95% CI: 95% Confidence interval
SD: Standard deviation
NICU: Neonatal intensive care unit
SD: Standard deviation
IVF: In vitro fertilisation
