# Supplementary material for: Metformin use and preeclampsia risk in women with diabetes: a two-country cohort analysis
Source: BMC Med. 2024 Sep 27;22:418. doi: 10.1186/s12916-024-03628-0 (PMC11438264; doi:10.1186/s12916-024-03628-0)
Supplement: Supplementary file 1 — Additional file 1: Statistical analysis plan. [file 12916_2024_3628_MOESM1_ESM.docx]

**Appendix**

**Table S1: Coding of covariates and outcome variables**

| **Clinical characteristic** | **Method of ascertainment** | **Description** |
| --- | --- | --- |
| Preeclampsia | ICD O11.1-5, O11.9 | Pre-existing hypertension with preeclampsia |
|  | ICD O14.0-2, O14.9 | Preeclampsia including HELLP syndrome |
|  | ICD O15.0-2, O15.9 | Eclampsia |
| Hypertensive diseases of pregnancy | ICD O13.1-5, O13.9 | Gestational (pregnancy induced) hypertension without significant proteinuria |
|  | ICD O11.1-5, O11.9 | Pre-existing hypertension with preeclampsia |
|  | ICD O14.0-2, O14.9 | Preeclampsia including HELLP syndrome |
|  | ICD O15.0-2, O15.9 | Eclampsia |
| Chronic hypertension | O10.0-4, O10.9 | Pre-existing hypertension complication pregnancy and the puerperium |
|  | ICD O11.1-5, O11.9 | Pre-existing hypertension with preeclampsia |
|  | ICD I10 | Essential (primary) hypertension |
|  | Check-box coding in the Swedish Medical Birth Register | Coded during antenatal care in response to “Chronic hypertension”, coded: “Yes” |
| Systemic lupus erythematosus (SLE) | Check-box coding in the Swedish Medical Birth Register | Coded during antenatal care in response to “SLE”, coded: “Yes” |
| Kidney disease | Check-box coding in the Swedish Medical Birth Register | Coded during antenatal care in response to “Kidney disease”, coded: “Yes” |
| Polycystic ovarian syndrome | ICD E28.2 | Polycystic ovarian syndrome |
| Gestational diabetes | ICD O24.4 | Gestational diabetes |
|  | Check-box coding in the Scottish Morbidity Records | In response to “Mother’s status of diabetes”, coded: “Yes, Gestational diabetes (diagnosed during pregnancy)” |
| Type 2 diabetes | E24.1 | Pre-existing type 2 diabetes mellitus, in pregnancy, childbirth and the puerperium |
|  | E11.0-9 | Type 2 diabetes mellitus |
| Congenital anomaly (neonatal) | Coded in the Scottish Morbidity Record | In response to “Congenital anomaly”, coded: “Acute life-threatening” “Non-life threatening” or “Yes” |
| Neonatal intensive care unit admission | Coded in Scottish Morbidity Record | In response to “Neonatal indicator”, coded: “Admitted for up to 48 hours” or “Admitted for more than 48 hours” |
| Neonatal death | Coded in Scottish Morbidity Record | In response to “Outcome of pregnancy”, coded: “Livebirth dying within the first six days of life” or “Livebirth dying on or after the 7^th^ day but before the 28^th^ day” |
| Body mass index | Scottish Morbidity Record | Calculated from maternal height and weight (weight (kg) / height (m^2^)) |
|  | Swedish Medical Birth Register |  |
| Smoking status | Coded in Scottish Morbidity Record | In reponse to “Smoker during pregnancy”, coded: “Yes” |
|  | Swedish Medical Birth Register |  |
| Socioeconomic status | Scottish Index of Multiple Deprivation | Recorded in quintiles |
| Maternal country of birth | Sweden Statistics | Coded as “Nordic countries”, “Europe & North America”, “Asia”, “Other” |
| Conception with in-vitro fertilisation | Check-box coding in the Swedish Medical Birth Register | In response to “IVF pregnancy”, coded: “Yes” |
| Highest level of maternal education | Education Register, Sweden Statistics | Coded as “University”, “Secondary school”, “<12 years of school attendance” |

Abbreviations: ICD = International Classification of Diseases

**Table S2: Covariate balance: Standardized differences**

|  | | **Scotland cohort** | | **Sweden cohort** | |
| --- | --- | --- | --- | --- | --- |
|  |  | **Raw** | **Weighted** | **Raw** | **Weighted** |
| **Maternal age >35 years** | | 0.16 | -0.09 | 0.07 | -0.11 |
| **Smoking during pregnancy** | | -0.01 | 0.17 | -0.05 | 0.06 |
| **Body mass index >30kg/m^2^** | | 0.21 | -0.14 | 0.48 | 0.03 |
| **Year of delivery** | | 0.84 | -0.02 | 0.78 | -0.07 |
| **Maternal education** | | N/A | | -0.11 | -0.04 |
| **IVF pregnancy** | | N/A | | 0.03 | -0.06 |
| **Country of birth** | | N/A | | 0.14 | 0.02 |
| **Pre-existing medical condition** (chronic hypertension, renal disease, systemic lupus erythematosus) | | N/A | | -0.01 | -0.01 |
| **Chronic hypertension** | | 0.03 | -0.05 | N/A | |
| **Polycystic ovary syndrome** | | 0.07 | -0.03 | N/A | |
| **Socioeconomic status (quintiles)** | **2** | 0.02 | 0.11 | N/A | |
|  | **3** | 0.05 | 0.01 |  |  |
|  | **4** | -0.10 | 0.04 |  |  |
|  | **5** | 0.00 | -0.03 |  |  |

**Table S3: STROBE Statement—Checklist of items that should be included in reports of *cohort studies***

|  | Item No | Recommendation | Page No |
| --- | --- | --- | --- |
| **Title and abstract** | 1 | (*a*) Indicate the study’s design with a commonly used term in the title or the abstract | 1 |
|  |  | (*b*) Provide in the abstract an informative and balanced summary of what was done and what was found | 4 |
| Introduction | | | |
| Background/rationale | 2 | Explain the scientific background and rationale for the investigation being reported | 6 |
| Objectives | 3 | State specific objectives, including any prespecified hypotheses | 7 |
| Methods | | | |
| Study design | 4 | Present key elements of study design early in the paper | 7 |
| Setting | 5 | Describe the setting, locations, and relevant dates, including periods of recruitment, exposure, follow-up, and data collection | 7 |
| Participants | 6 | (*a*) Give the eligibility criteria, and the sources and methods of selection of participants. Describe methods of follow-up | 7-8 |
|  |  | (*b*) For matched studies, give matching criteria and number of exposed and unexposed |  |
| Variables | 7 | Clearly define all outcomes, exposures, predictors, potential confounders, and effect modifiers. Give diagnostic criteria, if applicable | 9  Appendix |
| Data sources/ measurement | 8* | For each variable of interest, give sources of data and details of methods of assessment (measurement). Describe comparability of assessment methods if there is more than one group | Appendix |
| Bias | 9 | Describe any efforts to address potential sources of bias | 9, 20 |
| Study size | 10 | Explain how the study size was arrived at | 7 |
| Quantitative variables | 11 | Explain how quantitative variables were handled in the analyses. If applicable, describe which groupings were chosen and why | 10-11 |
| Statistical methods | 12 | (*a*) Describe all statistical methods, including those used to control for confounding |  |
|  |  | (*b*) Describe any methods used to examine subgroups and interactions |  |
|  |  | (*c*) Explain how missing data were addressed | 10-11 |
|  |  | (*d*) If applicable, explain how loss to follow-up was addressed |  |
|  |  | (*e*) Describe any sensitivity analyses |  |
| Results | | |  |
| Participants | 13* | (a) Report numbers of individuals at each stage of study—eg numbers potentially eligible, examined for eligibility, confirmed eligible, included in the study, completing follow-up, and analysed | 12  Figure 1 |
|  |  | (b) Give reasons for non-participation at each stage |  |
|  |  | (c) Consider use of a flow diagram |  |
| Descriptive data | 14* | (a) Give characteristics of study participants (eg demographic, clinical, social) and information on exposures and potential confounders | 12, 13 (Table 1) |
|  |  | (b) Indicate number of participants with missing data for each variable of interest |  |
|  |  | (c) Summarise follow-up time (eg, average and total amount) |  |
| Outcome data | 15* | Report numbers of outcome events or summary measures over time | 15  (Table 2) |

| Main results | 16 | (*a*) Give unadjusted estimates and, if applicable, confounder-adjusted estimates and their precision (eg, 95% confidence interval). Make clear which confounders were adjusted for and why they were included | 15  (Tables 2, 3) |
| --- | --- | --- | --- |
|  |  | (*b*) Report category boundaries when continuous variables were categorized |  |
|  |  | (*c*) If relevant, consider translating estimates of relative risk into absolute risk for a meaningful time period |  |
| Other analyses | 17 | Report other analyses done—eg analyses of subgroups and interactions, and sensitivity analyses | 14 |
| Discussion | | | |
| Key results | 18 | Summarise key results with reference to study objectives | 18 |
| Limitations | 19 | Discuss limitations of the study, taking into account sources of potential bias or imprecision. Discuss both direction and magnitude of any potential bias | 19-20 |
| Interpretation | 20 | Give a cautious overall interpretation of results considering objectives, limitations, multiplicity of analyses, results from similar studies, and other relevant evidence | 18-20 |
| Generalisability | 21 | Discuss the generalisability (external validity) of the study results | 20 |
| Other information | | | |
| Funding | 22 | Give the source of funding and the role of the funders for the present study and, if applicable, for the original study on which the present article is based | 2 |

*Give information separately for exposed and unexposed groups.

**Table S4: Relationship between metformin use (multiple scripts) and preeclampsia in Scottish nulliparous singletons with diabetes**

|  | **Multiple (≥2) metformin scripts filled (N=637)** | **Metformin unexposed**  **N= 2,672** | **Crude RR**  **(95% CI)** | **Inverse probability weighted RR (95% CI)** |
| --- | --- | --- | --- | --- |
| Hypertensive disorders of pregnancy | 43 (6.8) | 209 (7.8) | 0.86 (0.63-1.18) | 0.96 (0.61-1.49) |
| Preeclampsia | 18 (2.8) | 77 (2.9) | 0.98 (0.59-1.63) | 0.96 (0.47-1.95) |
| Preterm preeclampsia <37/40 | 4 (0.6) | 30 (1.1) | 0.47 (0.16-1.39) | 0.56 (0.18-1.73) |
| Pregnancy-induced hypertension | 25 (4.0) | 132 (5.1) | 0.79 (0.52-1.21) | 0.96 (0.53-1.75) |

**Additional File 1: Analysis plan for investigating associations between metformin use in pregnancy and preeclampsia among women with diabetes**

**Authorship team**

Dr Hannah Gordon, Prof Sue Walker, Dr Anthea Lindquist, Dr Manarangi De Silva, Prof Cathy Cluver, Dr Parinaz Mehdipour, Dr Richard Hiscock, Prof Stephen Tong, Dr Roxanne Hastie

**Research question**

Among women with diabetes, is metformin associated with a reduced likelihood of preeclampsia?

Main analysis

Overview

P–Nulliparous diabetic (gestational diabetes or Type II diabetes) women with a birth recorded in the Scottish maternity and neonatal birth record between 2009 and 2018

I–Prescription of metformin during pregnancy

C–No prescription of metformin during pregnancy

O–Hypertensive disorders of pregnancy

**Null hypothesis**

Taking metformin during pregnancy does not affect the likelihood of developing preeclampsia among women with diabetes.

1. **Background**

Metformin is an oral hypoglycaemic agent prescribed to women in pregnancy with pre-existing Type II diabetes and gestational diabetes. It has well-described benefits for mother and neonate when used in this context [22]. Beyond diabetes, metformin has been reported to reduce the likelihood of pregnancy complications among women with polycystic ovary syndrome [23, 24], and in women with previous unsuccessful attempts at in vitro fertilisation (IVF) [25].

Compared with insulin, two meta-analyses concluded that metformin administration is associated with lower rates of preeclampsia[22], and pregnancy-induced hypertension [8]. However, in women with diabetes, studies in this area have been small. Population-based research with larger numbers could generate stronger evidence to better investigate this relationship.

The objective of this research is to determine the relationship between metformin and hypertensive disorders of pregnancy (preeclampsia, eclampsia, gestational hypertension) among women with diabetes, using a national population-based cohort from Scotland.

1. **Study population**

The population will include all nulliparous singleton pregnancies with a diagnosis of diabetes (gestational diabetes or Type II diabetes), recorded in the Scottish Morbidity Records between 2009 and 2018. The Scottish Morbidity Records hold data relating to pregnancy, delivery and perinatal outcomes for all women and infants discharged from maternity hospitals across Scotland.

Exclusion criteria:

a) Multifetal pregnancy

b) Parous women (to account for clustering)

c) Missing gestational age (which will be used to calculate gestational age at exposure)

**Exposure**

Maternal metformin use during pregnancy will be identified through the Scottish National Prescribing Information System (PIS), which has been linked with the Scottish Morbidity Records. Metformin use will be further categorised as use ever, 0 – 12 weeks, 13 – 27 weeks, and 28 weeks onward. Metformin exposure will be assessed for timing, with only prescription prior to preeclampsia diagnosis included.

1. **Primary outcomes:**

Diagnosis of preeclampsia identified by codes O11, O14 and O15 according to the International Classification of Disease Tenth Revision.

Preeclampsia will be further classified by stratifying according to gestational age at birth; <37 weeks and <34 weeks.

Secondary outcomes:

- Gestational hypertension ICD-O13
- Neonatal birthweight: small for gestational age at birth or large for gestational age at birth (defined according to **1)** < or > 2 SD; adjusted for gestational age and offspring sex or **2)** birthweight centile calculated using GROW charts; <10^th^ percentile for SGA and >90^th^ percentile for LGA) (binary)
- Macrosomia; birthweight >4500g (binary)
- Neonatal intensive care unit admission (obtained via neonatal indicator variable)
- Major congenital abnormality (binary)
- Congenital renal anomaly (binary)
- Perinatal death (binary)
- Preterm birth

1. **Additional analyses:**

- Metformin exposure for any indication, compared to the total unexposed population (whether or not they were diagnosed with diabetes)
- It is anticipated that most women requiring metformin in pregnancy will have been prescribed this in the context of Gestational Diabetes or Type II diabetes. The main analysis has therefore been limited to diabetic women, to eliminate diabetes as a confounder
- By number of scripts filled for metformin (possibly reflects compliance)
- By dose, categorised as 1g and >1g

1. **Outline of statistical analysis**
   1. Descriptive analysis

Summary statistics of baseline characteristics of patients will be presented with mean (SD) used to describe normally distributed continuous variables, median (IQR) for skewed continuous variables, and n (%) for categorical variables. Univariate analyses will be performed for each of the variables of interest to be included in the model.

Population characteristics to be described for:

a) Overall cohort

b) Metformin exposed vs non-exposed cohort

| Characteristic | Descriptor | Total population | Metformin exposed | Metformin unexposed |
| --- | --- | --- | --- | --- |
| **Age,** (years) | mean ±SD |  |  |  |
| **Age >35 years** | n (%) |  |  |  |
| **Body mass index** (kg/m^2^) | mean ± SD |  |  |  |
| **Body mass index >30mg/kg** | n (%) |  |  |  |
| **Ethnicity** | n (%) |  |  |  |
| **Smoking,** yes | n (%) |  |  |  |
| **Deprivation score**  1  2  3  4  5 | n (%) |  |  |  |
| **Pre-gestational disorders**  Hypertension  Cardiovascular disease  Autoimmune diseases  Diabetes  Type I  Type II  Renal disease | n (%) |  |  |  |
| **Year of birth**  2009  2010  2011  2012  2013  2014  2015  2016  2017  2018 | n (%) |  |  |  |
| **Gestational diabetes** | n (%) |  |  |  |

- 1. **Outcome analysis**

Potential estimators are (i) selection model, using inverse probability weighting (IPW); (ii) outcome model, using regression adjustment; (iii) doubly robust inverse probability weighted regression adjustment (IPWRA) models. Under correct specification, all the estimators should produce similar results. We will focus on IPWRA; selection model will be included in the IPW and outcome in regression (RA) model specification. We will combine both regression adjustment and augmented IPW in a doubly robust regression model. We will also perform RA, IPW and multivariate LR separately.

Model for exposure assignment using inverse probability weighted propensity scores derived from the logistic exposure model. Adequacy of balance achieved by PS weighting will be assessed using: (i) standardised mean difference for model covariates and variance estimates between exposure groups and (ii) propensity scores overlap. If unbalanced, we will explore measures such as: addition of interaction terms; different exposure models; use of alternate weighting structures (overlap or stabilized); and lastly propensity score-based trimming with the aim of achieving overlap if the propensity score distributions.

This will be performed for the overall cohort and then subgroup analyses will be performed.

The model will be created for each outcome with the final included covariates determined by the authorship team and informed by direct acyclic graphs (Fig S1, S2). Covariates to be considered:

1. Maternal age at delivery
2. Body mass index
3. Deprivation score
4. Ethnicity
5. Smoking status
6. Pre-existing maternal disease
7. Conception with assisted reproduction

All statistical analysis will be performed using StataSE version 17.0.

- 1. Handling of missing data

a. Consideration of missing data will be confined to all the covariates identified for the construction of a (propensity score) model of the exposure status. When imputing these missing covariate values the imputation model will include all those identified for inclusion in the propensity score model as well as additional covariates. These co-variates will be selected once the data are available for review.

b. Multiple imputation will be performed using chained equations, and the imputed datasets will then be used for propensity score construction. Subjects with missingness greater than 50% of PS_covariates will be excluded from analysis, this proportion will be assessed and if substantial, may result in changes to the PS_covariate list used for ongoing analysis.

c Diagnostics will then be performed to assess the quality of the imputed datasets.

d. The number of imputed datasets will be set at a minimum of 20 with the number increased to equal to the highest percentage of missingness in the raw PS_covariate list (ceiling of 50% by design). For each of these imputed datasets IPWRA will be calculated and Rubin’s rule will be used pool parameters of estimate. Details of imputation and assessment of adequacy will be formally presented.

If imputation is unsuccessful, a complete case analysis will be used.
