# Supplementary figures and images for: Metformin use and preeclampsia risk in women with diabetes: a two-country cohort analysis
Source: BMC Med. 2024 Sep 27;22:418. doi: 10.1186/s12916-024-03628-0 (PMC11438264; doi:10.1186/s12916-024-03628-0)

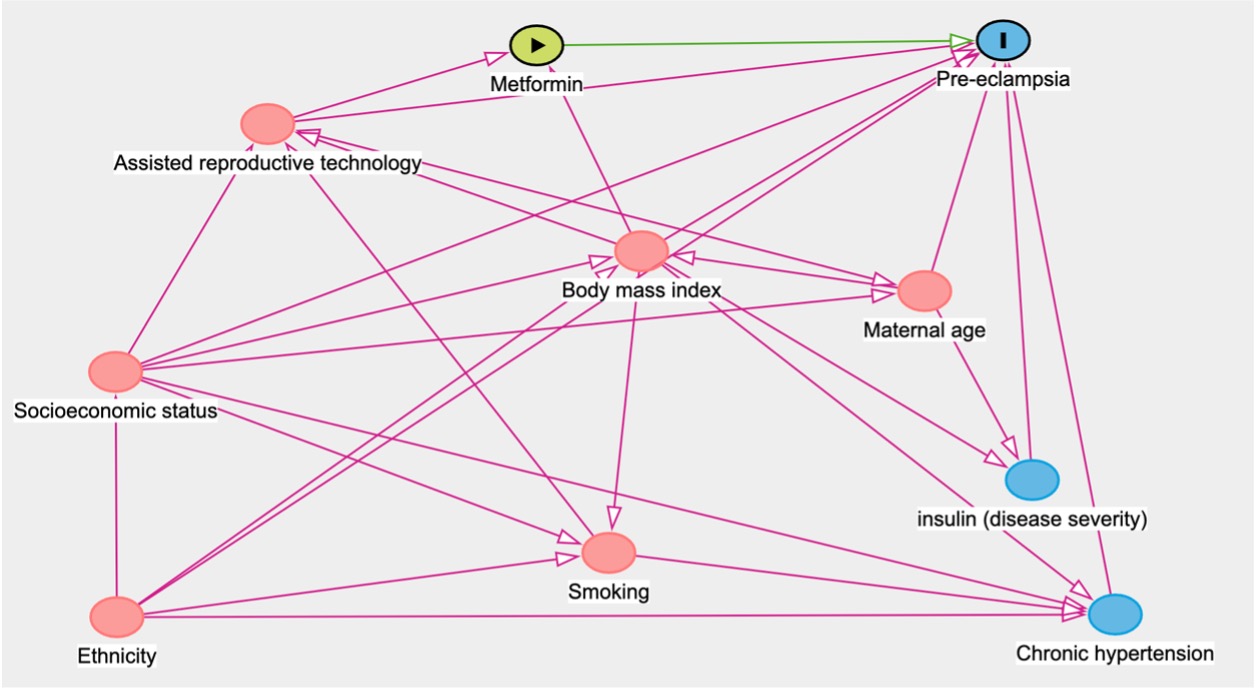

Supplement: Supplementary file 2 — Additional file 2: Figure S1 – Directed acyclic graph of the hypothesised relationship between metformin use in pregnancy and preeclampsia in the diabetic population. [file 12916_2024_3628_MOESM2_ESM.jpg]

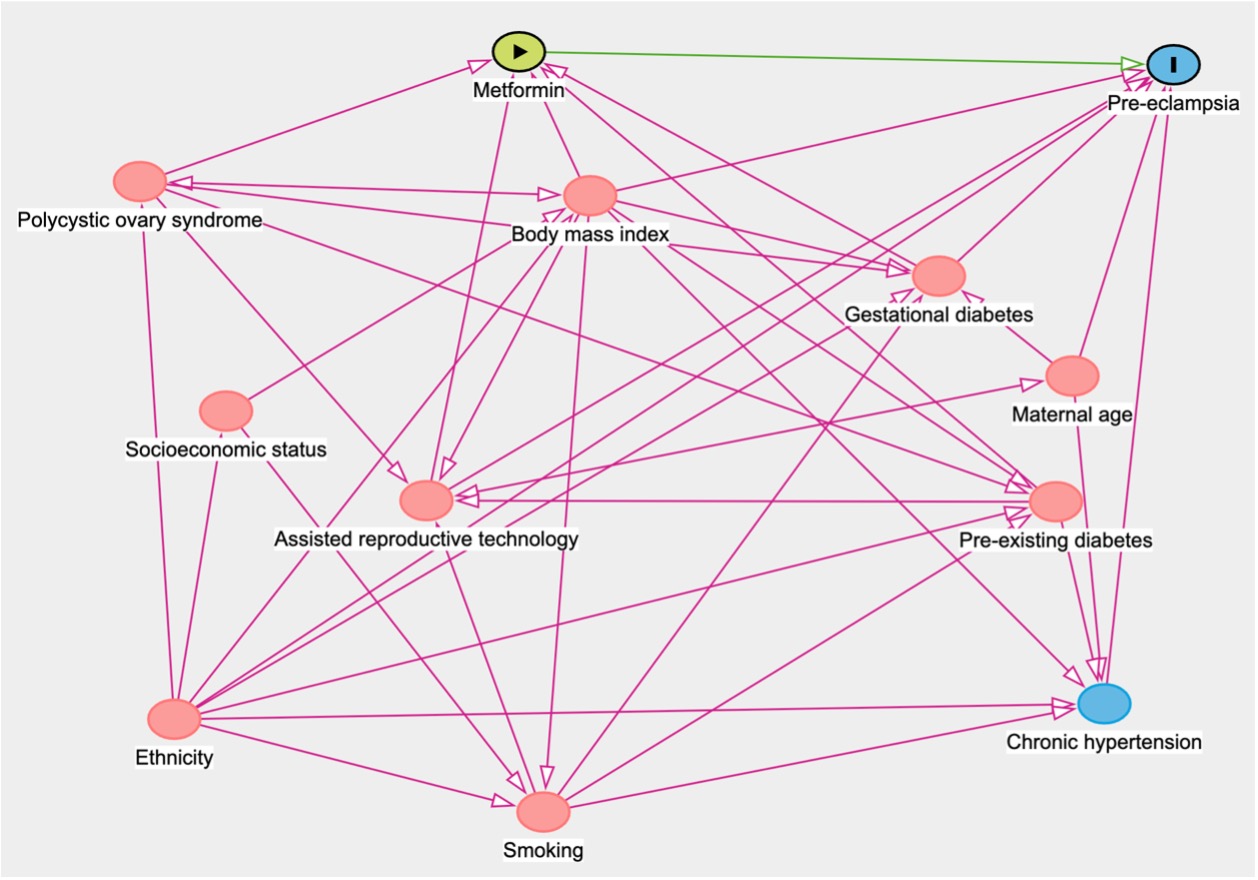

Supplement: Supplementary file 3 — Additional file 3: Figure S2 – Directed acyclic graph of the hypothesised relationship between metformin use in pregnancy and preeclampsia (irrespective of diabetes status). [file 12916_2024_3628_MOESM3_ESM.jpg]
